# Navigating the blood–brain barrier: enhancing blood culture practices in the neuro-ICU

**DOI:** 10.1017/ice.2024.235

**Published:** 2025-03

**Authors:** Maureen Metz, Katharine Colton, Jessica Seidelman

**Affiliations:** 1 Departments of Neurology, Duke University, Durham, NC, USA; 2 Division of Infectious Diseases and International Health, Department of Medicine, Duke University School of Medicine, Duke University, Durham, NC, USA; 3 Duke Center for Antimicrobial Stewardship and Infection Prevention, Duke University Medical Center, Durham, NC, USA

## Abstract

This study evaluates the implementation of a blood culture (BCx) algorithm in the neurology ICU (NICU) to reduce BCx event (BCE) rates. Results show a reduction in BCE rates, without increasing adverse outcomes. The findings support the feasibility of BCx algorithms for improving diagnostic stewardship in the specialized NICU population.

## Background

The liberal use of blood cultures (BCxs) can lead to patient harm through unnecessary antibiotic use, increased costs, and the risk of false-positive results.^1^ Successful implementation of BCx algorithms in other settings has demonstrated that reducing unnecessary cultures does not compromise patient care in the context of surgical intensive care units.^2^ However, the application of such algorithms in neurology ICUs (NICUs), a setting with unique challenges (eg central fever), has not been well studied. Our study aimed to determine whether implementing a BCx algorithm to determine when a BCx should be ordered could safely reduce the rates of BCx in this specialized population.

## Methods

This quasi-experimental, pre- and post-intervention study was conducted in the NICU of a single academic hospital from January 2022 to July 2024. We compared BCx rates before (01/2022–04/2023) and after (05/2023–06/2024) the implementation of a BCx algorithm using an interrupted time series. During the intervention period, we reviewed most BCxs and provided weekly feedback on appropriateness to clinicians and unit leadership as previously described by Seidelman et al.^2^ The study received approval from our hospital’s institutional review board (Pro00109734).

Patients were included if they had a BCx drawn while admitted to the NICU and ≥ 18 years of age. We defined a blood-culture event (BCE) as collection of a blood-culture set or blood-culture sets ordered by a clinician for a specific clinical indication. A BCE was inappropriate if the clinician did not follow the blood-culture algorithm. True-positive cultures were BCx that resulted in growth and were treated with antibiotics. Contaminants were unconventionally defined as BCx that resulted in growth and were not treated with antibiotics.

## Results

**Figure 1.**
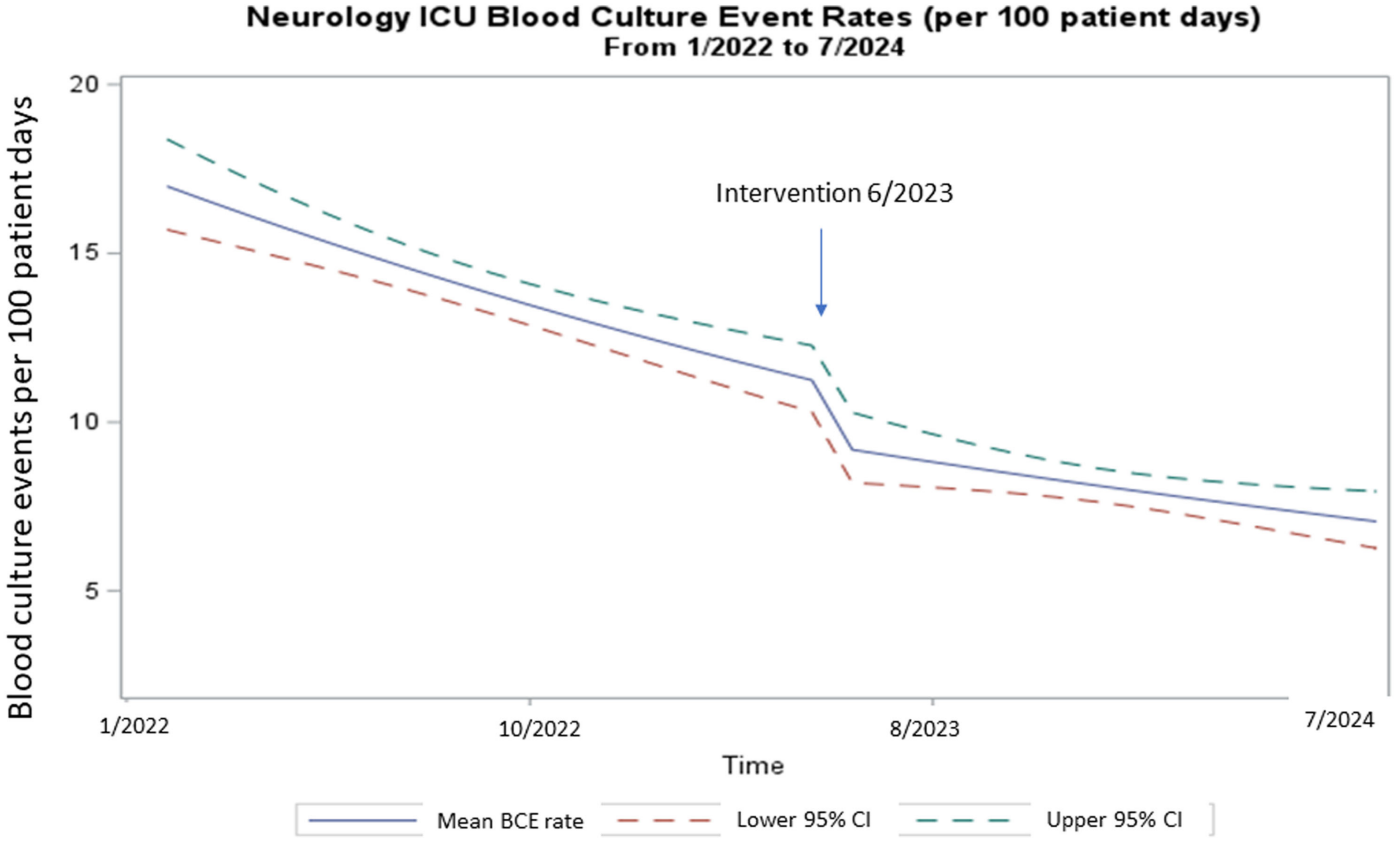
Interrupted time-series analysis of BCx algorithm implementation comparing the pre-intervention period (1/2022 to 5/2023) to the post-intervention period (6/2023 to 7/2024).

During the study period, we analyzed a total of 3,129 BCE: 2098 during the pre-intervention period and 1,031 in the post-intervention period. During the pre-intervention period, the BCE rate was decreasing by 2.6%. At the time of the intervention, the BCE rate decreased by 27.7%. Following the intervention, the rate continued to decrease by 0.5%. The incidence rate ratio pre-intervention compared to post-intervention was 0.56 (95% CI 0.51, 0.62, *P* < 0.01). (Figure 1)

We compared several adverse event outcomes during the pre-intervention versus post-intervention phases and found no significant increase (Table 1).

**Table 1.** Adverse outcome event comparison between the pre-intervention versus post-intervention phase

	Pre-intervention (1/2022-5/2023)	Post-intervention (6/2023-7/2024)	p-value
Average blood culture rate (per 100 admissions) (standard deviation)	14.0 (2.5)	8.1 (1.8)	**<0.01**
Monthly in-hospital mortality (standard deviation)	0.013 (0.036)	0.031 (0.068)	0.37
Monthly 30-day all-cause readmission (standard deviation)	0.84 (0.28)	0.60 (0.30)	**0.03**
Central line-associated bloodstream infection rate per 1000 line days (standard deviation)	2.28 (4.0)	1.0 (2.3)	0.17
Average monthly antibiotic days of therapy (standard deviation)	724 (134)	656 (106)	0.12

We reviewed a total of 298 BCx events from May 2023 to November 2023. The most common reasons for BCx were isolated fever and/or leukocytosis (134, 45%), severe sepsis or septic shock (46, 15%), and suspected infective endocarditis or endovascular infection (23, 8%). Two-hundred and fifty-four (85%) yielded negative cultures, 28 (10%) true positives, and 16 (5%) contaminant cultures. Of the 298 cultures reviewed, 158 (53%) were deemed to be inappropriate, 135 (45%) were adjudicated as appropriate, and 5 (2%) did not have enough information in the chart to make a delineation. Of the 158 inappropriate BCxs, the most common reasons for inappropriate BCxs were isolated fever and/or leukocytosis (134, 85%), ventilator-associated pneumonia, and postoperative fever within 48 hours (5, 3%). Of the 134 BCx drawn for isolated fever and/or leukocytosis, 126 yielded negative cultures and 8 yielded contaminants. We did not find any true-positive BCx that were drawn for isolated fever and/or leukocytosis.

We did a subgroup analysis on 44 BCx drawn on neurosurgical patients. In this population, 19 BCx were drawn for isolated fever and/or leukocytosis and 3 were drawn for postoperative fever within 48 hours of surgery. All 21 of these cultures were negative. In fact, in this group, we only had 2 positive BCxs, which were drawn in the same patient after brain death before organ transplantation.

## Discussion

We found that the implementation of a BCx algorithm in the NICU significantly decreased the BCE rate without corresponding increases in adverse effects. Importantly, we observed no rise in antibiotic days of therapy (DOT), central line-associated bloodstream infections (CLABSIs), 30-day readmission rates, or in-hospital mortality following the algorithm’s introduction. To our knowledge, this is the first study to report the use of a BCx algorithm specifically in an NICU setting.

Unnecessary BCxs lead to a cascade of negative outcomes. These include prolonged hospital stays, increased healthcare costs, a heightened risk of false positives, overuse of antibiotics, and a higher likelihood of antibiotic-related side effects and interactions.^3,4^ Furthermore, these unnecessary BCxs cause patient discomfort due to repeated blood draws, which can lead to anemia and subsequent transfusions. The burden extends to nursing staff and laboratory personnel, who spend significant time collecting and processing these specimens.

The NICU patient population presents a distinct challenge when it comes to BCxs, largely due to the prevalence of central fever and or noninfectious (reactive) leukocytosis. Central fever most commonly arises in patients suffering from brain tumors, intraventricular hemorrhage, subarachnoid hemorrhage, cerebral vasospasm, and traumatic brain injury and is thought to result from hypothalamic dysfunction and damage to brain thermoregulatory centers.^3,5^ Reactive leukocytosis is also a common occurrence post-seizure, which can be a complication of a subarachnoid hemorrhage or traumatic brain injury. These two groups are also prone to central fever.^4,6^

In addition, the NICU population also includes patients who present with altered mental status, stroke, coma, or other neurologic impairments that prevent effective communication. These patients are therefore unable to communicate whether they are having symptoms that are typical indicators of bacteremia. As a result, there is an increased likelihood of ordering BCxs to rule out bacteremia, even in the absence of clear clinical signs.

Our findings align with other studies that have implemented BCx algorithms in various hospital settings, including emergency departments and ICUs. For example, Theophanous et al. demonstrated that the implementation of a BCx algorithm in emergency department patients as a diagnostic stewardship intervention significantly reduced the number of unnecessary BCxs, leading to more targeted use of resources without compromising patient outcomes.^7^ Seidelman et al. also implemented a diagnostic stewardship intervention aimed at optimizing BCx utilization in two surgical ICUs, highlighting the effectiveness of algorithm-driven approaches in reducing BCx events and promoting more efficient patient care.^2^ Finally, Fabre et al. reported on the DISTRIBUTE study, which applied a diagnostic stewardship intervention among adult nonneutropenic inpatients, showing significant improvements in BCx use and reductions in false positives.^1^ These studies collectively support our findings, reinforcing the utility of BCx algorithms in reducing unnecessary tests while maintaining patient safety and enhancing antimicrobial stewardship.

Our study does have a few notable limitations. First, it is a single-unit, interrupted, unblinded time-series analysis and is accompanied by the limitations thereof. Second, we used historical NICU data as a control instead of comparing the results to another hospital unit during the same period. However, the NICU’s unique characteristics make direct comparisons with other units difficult. Additionally, we used a nonstandard definition of “contaminants” to increase the sensitivity of our algorithm to potential cases of bacteremia, acknowledging the complexity of our patient population.

In summary, our study demonstrates that the implementation of a BCx algorithm in the NICU can reduce unnecessary BCE while maintaining patient safety. Future studies should seek to replicate these findings in multicenter designs to improve generalizability. Our study highlights the importance of utilizing targeted interventions like BCx algorithms in complex, high-risk patient populations, offering a pathway toward more efficient, evidence-based care.
